# Assessment of Antioxidant Capacity and Putative Healthy Effects of Natural Plant Products Using Soybean Lipoxygenase-Based Methods. An Overview

**DOI:** 10.3390/molecules23123244

**Published:** 2018-12-07

**Authors:** Mario Soccio, Maura N. Laus, Zina Flagella, Donato Pastore

**Affiliations:** Department of the Sciences of Agriculture, Food and Environment, University of Foggia, Via Napoli 25, 71122 Foggia, Italy; maura.laus@unifg.it (M.N.L.); zina.flagella@unifg.it (Z.F.)

**Keywords:** antioxidant activity, lipoxygenase, LOX/RNO method, LOX–FL method, ORAC, TEAC, phytochemicals, Antioxidant/Oxidant Balance, blood antioxidant status

## Abstract

In the last decades, increasing demand of antioxidant-rich foods and growing interest in their putative role in prevention of degenerative diseases have promoted development of methods for measuring Antioxidant Capacity (AC). Nevertheless, most of these assays use radicals and experimental conditions far from the physiological ones, and are able to estimate only one or a few antioxidant mechanisms. On the other hand, the novel LOX/RNO and LOX–FL methods, based on secondary reactions between the soybean lipoxygenase (LOX)-1 isoenzyme and either 4-nitroso-*N*,*N*-dimethylaniline (RNO) or fluorescein (FL), may provide a more comprehensive AC evaluation. In fact, they are able to detect simultaneously many antioxidant functions (scavenging of some physiological radical species, iron ion reducing and chelating activities, inhibition of the pro-oxidant apoenzyme) and to highlight synergism among phytochemicals. They are applied to dissect antioxidant properties of several natural plant products: food-grade antioxidants, cereal and pseudocereal grains, grain-derived products, fruits. Recently, LOX–FL has been used for ex vivo AC measurements of human blood samples after short- and long-term intakes of some of these foods, and the effectiveness in improving serum antioxidant status was evaluated using the novel Antioxidant/Oxidant Balance (AOB) parameter, calculated as an AC/Peroxide Level ratio. An overview of data is presented.

## 1. Introduction

Antioxidants are compounds able to respond in a different manner to different radical or oxidant sources [1] and include free radical scavengers, singlet oxygen quenchers, inactivators of peroxides and other reactive oxygen species (ROS), metal ion chelators, quenchers of secondary oxidation products, inhibitors of pro-oxidative enzymes [2], as well as compounds able to induce upregulation of antioxidant and detoxifying enzymes and modulation of redox cell signaling and gene expression [3]. During the last few decades, naturally occurring antioxidants have received increasing attention, especially within biological, medical and nutritional fields, owing to their putative protective roles against the deleterious oxidative-induced reactions implicated in food deterioration, as well as in the pathogenesis of several human diseases, such as atherosclerosis, diabetes mellitus, chronic inflammation, neurodegenerative disorders and certain types of cancer [2,4,5,6]. In the light of this, natural and potent antioxidants are in demand for food preservatives and nutraceuticals/pharmaceuticals. The effective search for sources of naturally occurring antioxidants and the design of novel antioxidant compounds require reliable methods of Antioxidant Capacity (AC) evaluation [2].

For this purpose, a considerable number of chemical assays, coupled with highly sensitive, quick and usually automated detection technologies, have been developed (Table 1) [1,7,8,9,10,11,12,13,14,15,16,17,18,19]. These assays may endow different information about the ROS–sample interaction, claiming to ensure a fast, simple, convenient, and reliable in vitro AC determination. In particular, they usually employ a chemical system containing an oxidant (free radicals or other ROS), an oxidizable probe (not necessary for some assays) and antioxidants under investigation.

Antioxidant activities may be either expressed as inhibition against ROS-mediated oxidation of the probe, or equivalents of a selected reference antioxidant such as Trolox, ascorbic acid or other compounds [2]. Depending upon the reactions involved, these assays can roughly be classified into two types: assays based on hydrogen atom transfer (HAT) reactions and assays based on single electron transfer (SET) [7] (see also Table 1). The HAT reaction, which is the most relevant to human biology [1], consists in a concerted movement of a proton and an electron in a single kinetic step, in which the free radical removes one hydrogen atom from an antioxidant and the antioxidant itself becomes a radical. In SET mechanisms, the antioxidant reduces the free radical by a single electron transfer. In most situations, HAT and SET reactions take place simultaneously and the reaction mechanism is determined by the antioxidant’s structure and solubility, the partition coefficient and solvent polarity [20]. 

Really, these methods show some technical limitations mostly related to the chemistry behind the different assays. In fact, most of these methods are able to evaluate the scavenging capacity of antioxidants only against specific types of radical species, some of which are not physiological and biologically irrelevant, failing to evaluate other important antioxidant effects. Therefore, an adequate combinatory application of AC assays showing different chemical basis could represent a more valid strategy for a more biologically relevant AC assessment.

Nevertheless, it should be outlined that in vitro AC determinations remain rather questionable for the difficulty in reflecting the in vivo situation [2,7,21,22]. In vitro chemical methods could not be enough to obtain reliable information on potential of dietary antioxidant as health-promoting agents. In fact, these chemical assays do not consider relevant parameters involved in biological environments, such as lipophilicity and bioavailability, in vivo stability, retention of antioxidants by tissues as well as reactivity in situ. Finally, there is growing evidence that the metabolic pathways associated with the prevention or amelioration of chronic diseases by bioactive compounds are often dependent on enzyme/protein and/or gene expression regulation rather than a true antioxidant effect [23,24]. For these purposes, AC assays have also been extended from food model systems to biological samples, cell lines, and even live tissues [2].

## 2. Methods Based on the Use of the Soybean Lipoxygenase-1 Isoenzyme: Lipoxygenase/4-Nitroso-*N*,*N*-Dimethylaniline (LOX/RNO) and Lipoxygenase–Fluorescein (LOX-FL) Assays

In order to develop innovative methodologies/approaches able to provide AC values as much as possible reflecting the in vivo response, two advanced assays, based on the use of soybean lipoxygenase (LOX)-1 isoenzyme as a system to generate different physiological radicals and to highlight different antioxidant mechanisms, have been developed. They were named LOX/RNO [25] and LOX–FL [26] methods, as they are based on the reaction between LOX-1 isoenzyme and 4-nitroso-*N*,*N*-dimethylaniline (RNO) [27] and that between LOX-1 isoenzyme and fluorescein (FL), respectively. The firstly developed LOX/RNO method was applied to dissect antioxidant properties of several natural products: food-grade antioxidants, cereal and pseudocereal grains, grain-derived products, and fruits [28,29,30,31,32]. By merging the advantages of LOX/RNO method, deriving from the use of soybean LOX-1 isoform, and the high sensitivity of ORAC assay due to use of FL as a probe [33], LOX–FL assay has been recently developed [26]. This has the added dimension with respect to LOX/RNO method to be a suitable tool for both in vitro measurements of food extracts and ex vivo analysis of human blood samples. In the light of this, it has been applied mainly to ex vivo AC measurements of human blood after both short-term [34] and long-term [35] intakes of food antioxidants. 

An up-to-date overview of data obtained from in vitro and ex vivo measurements using LOX-1-based assays is presented. 

### 2.1. Aerobic and Anaerobic Reactions Catalyzed by Soybean Lipoxygenase (LOX)-1 Isoform and Involvement of LOX-1-Mediated Reactions in RNO Bleaching and FL Quenching 

Lipoxygenases (linoleate: oxygen oxidoreductase, EC 1.13.11.12) (LOXs) are a large family of non-heme, non-sulfur iron-containing fatty acid dioxygenases, which occur ubiquitously in plants and mammals [36,37,38,39]. These enzymes catalyze the region- and stereo-specific insertion of molecular oxygen into polyunsaturated fatty acids containing at least one 1,4-*cis*,*cis* pentadiene moiety (e.g., linoleic, linolenic, and arachidonic acids) to produce the corresponding hydroperoxy derivatives [40]. LOXs play several physiological roles [41,42,43]. In particular, fatty acid hydroperoxides generated by plant LOXs can be metabolized into volatile aldehydes and jasmonates, playing a critical role as signal molecules in wound healing and defence processes [44]; in mammals, hydroperoxides are precursors of lipoxins and leukotrienes involved in inflammation, asthma and heart disease [45,46]. 

**Scheme 1:**
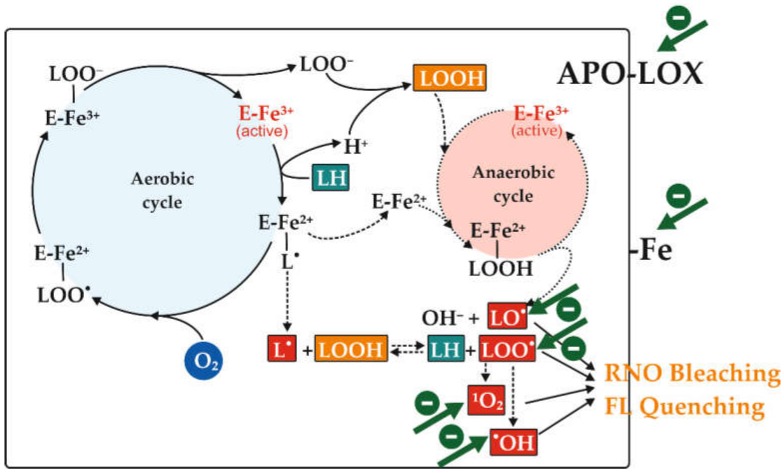
Aerobic and anaerobic reactions catalyzed by the soybean lipoxygenase (LOX)-1 isoenzyme and involved in 4-nitroso-*N*,*N*-dimethylaniline (RNO) bleaching or fluorescein (FL) quenching. The aerobic cycle of LOX-1-catalyzed dioxygenation of linoleic acid (LH, highlighted in teal) to 13-hydroperoxy-linoleate (LOOH, highlighted in orange) is shown (continuous line), as well as the secondary anaerobic cycle (dotted line) involving LOX-1 under limited oxygen conditions and generating some reactive species (highlighted in red). These may induce RNO bleaching or FL quenching. This may be inhibited by antioxidant compounds by different mechanisms (green arrows): scavenging one or more radical species; chelating or reducing iron ion; inhibiting the apoenzyme. L**^∙^**, linoleate alkylic radical; LO**^∙^**, linoleate alkoxyl radical; LOO**^∙^**, linoleate peroxyl radical; ^1^O_2_, singlet oxygen; **^∙^**OH, hydroxyl radical. “Fe” indicates the non-heme iron atom playing a key role in LOX-1 catalysis. Adapted from Pastore et al. [25].

The four consecutive reactions of linoleic acid (LH) dioxygenation to 13-hydroperoxy-linoleate (LOOH) catalysed by the soybean LOX-1 isoenzyme are shown in Scheme 1. A key role in LOX-1 catalysis is played by non-heme iron atom (Fe), cycling from the oxidized form (III) to the reduced one (II) [47]. The first step involves LH oxidation via stereo-selective hydrogen abstraction accompanied by reduction of non-heme ferric (III) iron to its ferrous (II) form; this leads to both proton release and generation of the linoleate alkylic (L**^∙^**) radical within the substrate-binding pocket of LOX-1–iron (II) complex. After alkylic radical rearrangement, molecular dioxygen insertion can occur generating the peroxyl radical (LOO**^∙^**)–enzyme–iron (II) complex. In the subsequent step, the peroxyl radical is reduced to the corresponding anion (LOO^–^) by one electron transfer from the ferrous (II) iron atom that is reoxidized to its ferric (III) form. Finally, the peroxyl anion is protonated to hydroperoxide (LOOH) and released from the enzyme–iron (III) complex, which can start a new cycle. When the main aerobic cycle consumes oxygen in the reaction mixture, the alkylic (L**^∙^**) radical reacts with LOOH generated during the aerobic cycle to form LH and LOO**^∙^ [48]**. The peroxyl radical can in turn generate hydroxyl radical (**^∙^**OH); moreover, it can lead to the formation of carbonyl compounds (dienals and oxodienes) [48] and singlet oxygen (^1^O_2_) through the Russell’s mechanism [49]. On the other hand, the enzyme–iron (II) can convert LOOH into hydroxyl anion (OH^–^) and linoleate alkoxyl (LO**^∙^**) radical by means of a Fenton-like reaction [47]. All these radicals are known to induce plant pigment oxidation with consequent bleaching [41]. Moreover, some of these reactive species (LO**^∙^**, LOO**^∙^**, **^∙^**OH and ^1^O_2_, but only in the presence of imidazole [50]) have been demonstrated to cause the bleaching of RNO in a biochemical pathway coupled with oxodiene formation [27]. Recently, the capability of these oxidant species to induce the bleaching and quenching of FL has been also demonstrated [26]. Interestingly, as shown in Scheme 1, both the soybean LOX-1-catalysed RNO bleaching (LOX/RNO reaction) and FL quenching (LOX–FL reaction) may be delayed, inhibited or even prevented by antioxidants acting according to different mechanisms (indicated by green arrows). In particular, these include the primary chain-breaking capacity of antioxidants to scavenge one or more free radical species, as well as secondary antioxidant mechanisms involving chelating or reducing activities of iron ion essential for LOX-1 catalysis, singlet oxygen quenching, hydroperoxide decomposition, and direct inhibition of pro-oxidative LOX-1 apo-enzyme. Therefore, with respect to the majority of AC assays, the peculiarity of LOX/RNO and LOX–FL reactions is to simultaneously detect, under conditions of low oxygen supply (as it often occurs in vivo), the primary scavenging capacity towards several different and biologically relevant radical species together with other important secondary antioxidant functions [25,26]. Thus, methods based on LOX/RNO or LOX–FL reactions may provide a more integrated and comprehensive information about AC of foods [25,26].

It should be outlined that, among methodologies for AC assessment of food antioxidants, assays involving inhibition of soybean LOX-induced fatty acid peroxidation have been already proposed [51,52,53]. Unlike LOX/RNO and LOX–FL reactions, these assays mainly evaluate secondary antioxidant mechanisms including the capability to prevent or retard lipid peroxidation by decomposing or inactivating lipid hydroperoxides, chelating iron ions, or directly inhibiting the pro-oxidative LOX enzyme. Interestingly, an assay based on β-carotene bleaching coupled to soybean LOX-induced linoleic acid peroxidation has been also developed [54]. While LOX/RNO and LOX–FL reactions have the advantage to use cheap and highly water-soluble probes for monitoring the reaction progress, the β-carotene-linoleic acid co-oxidation assay employs the highly fat-soluble β-carotene as a probe. Moreover, it should be considered that β-carotene itself is even an inhibitor of LOXs [55]; hence, this can lead to misleading results. Finally, LOX/RNO and LOX–FL reactions are able to highlight scavenging mechanisms of antioxidants against a series of biologically relevant oxidant species generated by soybean LOX-1 under low oxygen concentrations, while the inhibition of LOX-mediated β-carotene bleaching essentially depends on scavenging of peroxyl radicals produced during the aerobic cycle of linoleate oxidation [56]. 

### 2.2. LOX/RNO and LOX–FL Reactions: Main Kinetic Properties 

**Figure 1:**
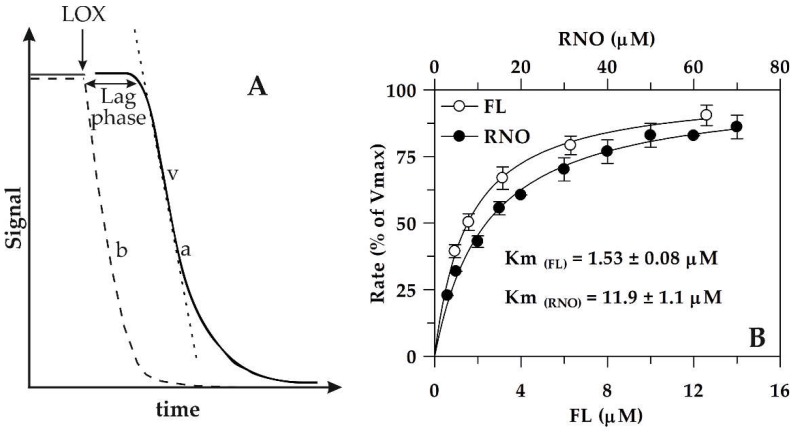
Kinetics of RNO bleaching and FL quenching catalyzed by soybean LOX-1. (**A**) Typical experimental traces are reported relative to: (i) spectrophotometric (or fluorimetric) measurement of the LOX-1-dependent RNO bleaching (or FL quenching) reaction (trace a) and (ii) simultaneous polarographic measurement of LOX-1-catalyzed oxygen uptake (dotted line, trace b). The reaction rate (v) and the lag phase are also indicated. (**B**) The rates of RNO bleaching and FL quenching, expressed as (%) of Vmax, are reported as Michaelis-Menten plots. The values of 100% Vmax correspond to 0.27 ± 0.10 ΔA_440_∙min^−1^ and 0.28 ± 0.01 ΔA_485_∙min^−1^ for LOX/RNO and LOX–FL reactions, respectively. Km values are also reported. Data are recalculated from those reported in Pastore et al. [25] and Soccio et al. [26] and expressed as mean value ± standard deviation (*n* = 3).

LOX/RNO reaction can be easily photometrically measured by continuously monitoring the decrease of signal, represented by RNO absorbance at 440 nm. Similarly, LOX–FL reaction may be followed as FL fluorescence decreases (λex = 485, λem = 515 nm). In Figure 1A, the typical progresses of LOX/RNO or LOX–FL reaction (trace a) and of simultaneous oxygen uptake measurement (dotted line, trace b) are schematically represented. 

As it is shown, the addition of LOX-1 to the reaction mixture containing excess linoleate and the limited oxygen concentration induces a rapid oxygen consumption (trace b in Figure 1A) due to linoleate dioxygenation. On the contrary, RNO bleaching or FL quenching, indicated by a rapid signal decrease as a function of time (trace a in Figure 1A), starts only after a lag phase ranging from about 20 to 120 s [25,26]. It represents the time necessary for the primary aerobic LOX-1-mediated LH peroxidation to reduce the oxygen concentration in the reaction mixture to 20–50 μM [27], so as to trigger secondary anaerobic reactions generating radical species able to induce signal decrease (see also Scheme 1).

The rate of LOX/RNO or LOX–FL reaction is calculated as the highest slope of the experimental trace. The rate of LOX/RNO reaction was reported to be dependent on LOX-1 amount, linoleate concentration, pH, and temperature, as well as sensitive to the powerful LOX inhibitor *n*-propylgallate [27]. Moreover, the enzymatic nature of both LOX/RNO and LOX–FL reactions was demonstrated by Pastore et al. [25] and Soccio et al. [26], who reported an hyperbolic dependence of reaction rate on RNO or FL concentration, respectively, as predicted by the Michaelis–Menten equation (Figure 1B), as well as by Lineweaver–Burk, Eadie–Hofstee, Eadie-Scatchard and Hanes plots. The occurrence of saturation dependence of reaction rate on RNO or FL concentration indicates that reactions does not occur in the bulk phase of reaction mixture, but at the level of LOX-1 substrate-binding pocket, generating a ternary enzyme–radical–RNO (or FL) complex. The property to occur in the body of a biological macromolecule is a very interesting prerogative of LOX/RNO and LOX–FL reactions, which can allow studying the effect of antioxidants according to a more physiological approach than the most widely used assays [25,26].

### 2.3. Inhibition of LOX/RNO and LOX–FL Reactions by Pure Antioxidant Compounds

**Figure 2:**
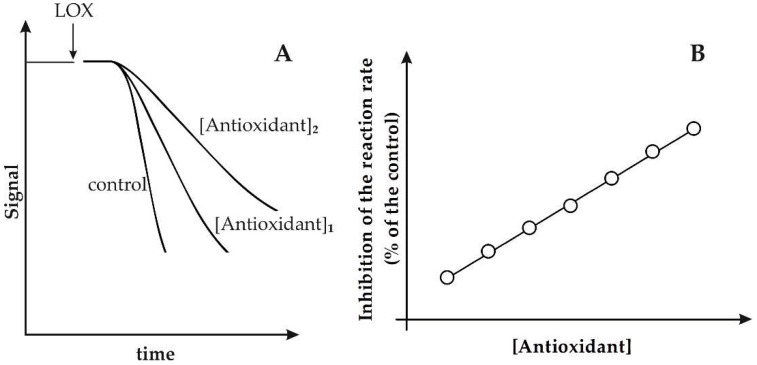
Inhibition of LOX/RNO (or LOX–FL) reaction by antioxidants. (**A**) Experimental traces relative to LOX/RNO (or LOX–FL) reactions measured in both the absence (control) and presence of two increasing concentrations of a generic antioxidant are shown. (**B**) The inhibition, expressed as (%) decrease of LOX/RNO (or LOX–FL) reaction rate with respect to the control, is reported as a function of antioxidant concentration.

In Figure 2A, the typical behavior of LOX/RNO or LOX–FL reaction in the presence of a generic pure antioxidant compound is reported. The capability of antioxidant to inhibit the LOX-1-based reactions by typically inducing a decrease of the reaction rate, with an increasing inhibition with an increasing amount of antioxidant, is clearly highlighted.

However, it should be also underlined that some antioxidants may significantly affect both the reaction rate and lag phase or may induce mainly an increase of lag phase [25,28]. The different behaviors of different antioxidant compounds with respect to both the reaction rate and lag phase can indicate different antioxidant actions of phytochemicals. A main radical scavenging activity may be suggested for compounds mainly inhibiting the reaction rate, while the capability of antioxidants to exert an evident effect on the lag phase may indicate an inhibition of LOX-1 hydroperoxidative activity (antiperoxidative action) [25,28]. Interestingly, as reported in Figure 2B, a linear plot can be obtained by reporting the antioxidant-dependent inhibition, expressed as (%) decrease of the reaction rate with respect to the control, as a function of antioxidant concentration. This allowed obtaining easily a proper calibration curve for both LOX/RNO and LOX–FL reactions using standard pure antioxidants. In particular, using Trolox, an α-tocopherol analogue with enhanced water solubility, calibration curves have been obtained for both LOX/RNO and LOX–FL reactions, described by the equations: (%)inhibition = 5.838 [Trolox](mM) + 11.374 (*r* = 0.996 ***) and (%)inhibition = 1.920 [Trolox](μM) + 3.23 (*r* = 0.996 ***), respectively [25,26].

Several pure compounds, belonging to the main classes (phenolic acids, flavonoids, stilbenes, tocols, carotenoids, vitamins, etc.) of antioxidants and differing for chemical/physical (hydrophilic and lipophilic) properties, were investigated with respect to the capability to affect LOX/RNO reaction [25]; moreover, the most representative endogenous antioxidants contained in human serum were tested on LOX–FL assay [26]. All tested compounds were found to affect LOX/RNO and/or LOX–FL assay by inducing an inhibition of reaction rate with different mechanisms and effectiveness. Nature of inhibition with Ki and/or IC_50_ values were determined for each antioxidant and the most relevant results are summarized in Table 2.

Among different antioxidant compounds, Trolox was found to inhibit LOX/RNO and LOX–FL reactions in a noncompetitive and competitive manner with a Ki value of about 7 mM and 5 μM, respectively [25,26]. Moreover, IC_50_ with a value equal to 26 μM for LOX–FL reaction was also obtained. With the only exception of β-carotene, a general lower sensitivity of LOX/RNO reaction to single pure antioxidants with respect to LOX–FL was observed, as indicated by millimolar and micromolar ranges of Ki or IC_50_ values obtained, respectively. Similarly to Trolox, all other investigated antioxidants showed a linear dependence of inhibition (%) of the reaction rate on compound concentration. In the light of this, comparison of efficacy of each compound with respect to Trolox was made (Table 2, column Antioxidant/Trolox) by calculating the ratio between the gradient of the plot relative to the antioxidant and that relative to Trolox. As for LOX/RNO reaction, resveratrol, apigenin and α-tocopherol displayed higher activity than Trolox, ferulic and l-ascorbic acids similar activity, while gallic acid and catechin resulted less active. Regarding LOX–FL reaction, bilirubin resulted more active than Trolox, uric and l-ascorbic acids showed lower activity, and similar activity was found for albumin.

### 2.4. Suitability of the LOX-1-Based Assays to Assess AC of Plant Food Extracts and Blood Samples

#### 2.4.1. Assessment of AC of Plant Food Extracts

In addition to pure antioxidant compounds, to date LOX/RNO and LOX–FL methods have been applied to AC evaluation of a wide variety of plant food matrices. Their performances have been always compared to that of two different well-established AC assays, such as ORAC and TEAC, measuring mainly the scavenging capacity against peroxyl and ABTS^∙+^ radicals according to SET and HAT mechanisms, respectively (see also Table 1). The main results are summarized in Table 3.

Firstly, the LOX/RNO assay has been applied to dissect AC of whole grains of durum wheat [25,28]. In the last few decades, in fact, cereal whole grains have received growing interest due to their unique and peculiar content in biologically active compounds, which are thought responsible of some beneficial health properties associated to regular grain consumption, including protection against chronic diseases and diet-related disorders (for a recent review, see Masisi et al. [62]). In particular, the LOX/RNO method highlighted significant differences among the different antioxidant-enriched extracts from durum wheat grains, with the highest AC values in phenolic fraction linked to insoluble cell wall polymers, which is known as the main antioxidant component in cereal grains [63]. Moreover, with respect to TEAC assay, the LOX/RNO method resulted able to better discriminate among samples obtained from different cultivars and under different experimental conditions (including different fertilizations and irrigation treatments), as well as providing a reliable AC information highly correlated to content in antioxidant compounds [28]. In comparison with durum wheat, LOX/RNO assay has been used also to evaluate AC of different extracts obtained from grains of emmer [29], bread wheat, spelt, hulled and naked einkorn [57,58]. Interestingly, unlike TEAC and ORAC assays, LOX/RNO was able to highlight remarkable differences among the examined cereal species in relation to the balance between free and bound grain antioxidants, with a clear superiority of the hulled cereal species for both AC of insoluble-bound phenolic component and AC of freely solvent-soluble antioxidants. This may suggest a deeply different potential action of antioxidants on the health of consumers, since free compounds may exert a better systemic healthy activity, while bound antioxidants may have a useful activity at the level of the terminal intestine [64,65,66]. Antioxidant properties of teff and finger millet grains were also measured by the LOX/RNO method [59,60,61], as well as AC of the pseudocereals quinoa [29], amaranth and buckwheat [59,60,61]. In the case of quinoa, with respect to the other compared AC assays, LOX/RNO was once again able to unearth very interesting antioxidant properties, potentially related to health benefits. In particular, LOX/RNO pointed out a much higher AC of the freely soluble and more readily accessible antioxidant fraction of quinoa seeds than that of whole grains of the traditionally consumed cereals durum wheat and emmer, thus increasing interest for utilization of quinoa-derived products, not only for celiac diet, but also for nutrition of general population [29].

AC of some cereal-grain-derived products was also studied by both LOX-1-based assays. In particular, antioxidant properties of Lisosan G, an antioxidant-rich dietary supplement obtained from lysed fine bran and germ of organic wheat grains with a well-documented bioactivity [67,68,69,70], were studied by both LOX/RNO [30] and LOX–FL methods [26]. In accordance with high antioxidant properties of fermented whole grain products due to the improved content of water-soluble compounds [62], both LOX-1-based methods showed a remarkable total AC of Lisosan G. It resulted from 3- to 10-fold higher than that measured for different cereal species, and it is mainly attributable to a very active freely water-extractable antioxidant component together with insoluble-bound phenols [26,30].

Among cereal-grain-derived products, very high AC values were also measured by both LOX/RNO and LOX–FL assays in a hydrophilic/phenolic antioxidant-rich extract obtained by ultrasound-assisted water extraction of durum wheat bran (bran water extract, BW extract). In addition, LOX–FL assay was used to dissect AC of two cooked semolina pastas, supplemented with Bran Oleoresin (BO) extract rich in lipophilic (tocochromanols, carotenoids) antioxidants and with BW extract, in comparison with a non-supplemented pasta [34]. Interestingly, in agreement with the different supplementation of BW and BO pastas, LOX–FL highlighted the highest AC values in the free-soluble phenolic and lipophilic extracts of BW and BO pastas, respectively, while contrasting results were provided by ORAC and TEAC [34].

It should be also outlined that LOX/RNO proved to be an excellent tool for AC assessment of food-grade preparations obtained from very different plant sources and highly enriched in either lycopene or different phenolic compounds, including the flavonoids catechins and quercetin and the non-flavonoid resveratrol and tyrosol/hydroxytyrosol/oleuropeine mixture (OLIPLUS^®^) [31].

Good performance was also obtained by LOX-1-based assays in evaluating AC of fruits and vegetables. In particular, in carotenoid-enriched extracts from peach fruits, the LOX/RNO method measured AC values highly related to carotenoid content, and pointed out a much higher differences among yellow- and white-fleshed varieties than ORAC and TEAC methods [32]. Moreover, the free-soluble phenolic component obtained from cherry puree [35], as well as of pomegranate fruit juice and hydrophilic and lipophilic fractions from tomato fruit, was also evaluated by LOX–FL assay. Finally, LOX–FL assay has been applied to assess antioxidant properties of very complex matrices, such as extra-virgin olive oil and red wine.

In conclusion, LOX-1-based assays proved to be advisable tools for measurement of antioxidant potential of a large variety of plant foods, showing the capabilities of easily discriminating among different samples and of providing a reliable AC information correlated to antioxidant content and possibly related to health benefit.

#### 2.4.2. Assessment of AC of Blood Samples

**Figure 3:**
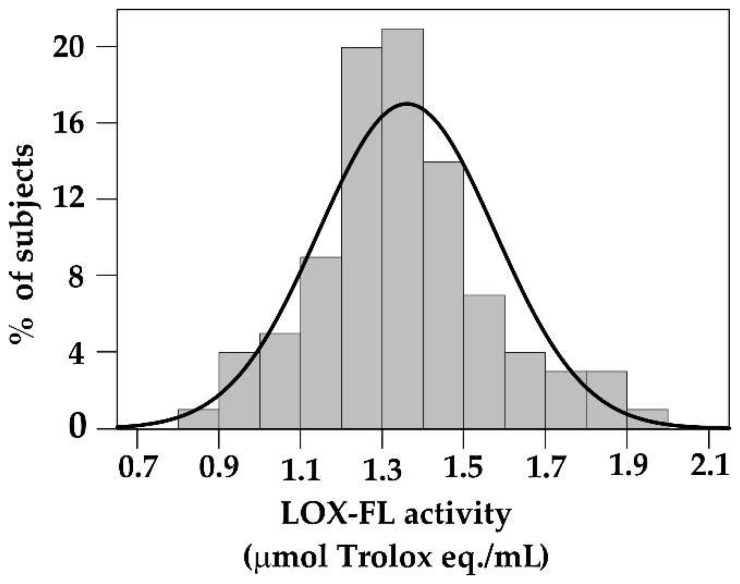
Distribution of serum Antioxidant Capacity values obtained by LOX–FL assay. Serum AC values were obtained from 92 subjects after an overnight fast.

As already reported (Section 2), in addition to being used for AC measurements of food extracts, LOX–FL assay has the added dimension with respect to the LOX/RNO method to be a suitable tool also for analysis of biological samples, such as human blood samples [26]. In fact, LOX–FL method can be used on both human blood serum and plasma, while LOX/RNO assay was found to require a too high volume of both biological fluids. However, caution has to be paid when plasma is obtained using EDTA or citrate as anticoagulants, since they may chelate iron that is necessary to LOX activity. In the light of this, LOX–FL assay has been preferably applied to ex vivo AC assessment of human blood serum. To date, a large number of serum samples has been analyzed by LOX–FL reaction. A normal distribution of AC data was obtained within a very wide range between 0.8 and 2 μmol Trolox eq./mL of serum (Figure 3). Interestingly, for the property to be applied to both in vitro and ex vivo AC measurements, LOX–FL assay can play an important role also in the second level of investigation, involving AC analysis of serum/plasma after food intake. High performance of LOX–FL method in both in vitro and ex vivo measurements has the advantage to provide an integrated AC evaluation of food antioxidants: by using the same assay, it is possible to verify if foods showing strong AC may provide a real beneficial effect after ingestion.

### 2.5. Evaluation of Synergistic Effects Among Antioxidants Using LOX-1-Based Assays

LOX-1-based AC assays were demonstrated to reveal very well synergism among antioxidant compounds. Table 4 shows results of different experiments aimed at evaluating the capability of both LOX/RNO and LOX–FL reactions, in comparison with ORAC and TEAC assays, to highlight antioxidant synergistic interactions.

By comparing AC of the mixture of compounds with the sum of AC of individual compounds, the LOX/RNO method showed an about 20 times higher ability, compared to TEAC, to assess synergistic interactions among food-grade preparations highly enriched in phenolic compounds, including catechins, quercetin, resveratrol, and tyrosol/hydroxytyrosol, as well as in carotenoids such as lycopene [31]. Similarly, also LOX–FL assay proved able to measure synergistic effects among the most representative antioxidants of human blood serum, including ascorbic and uric acids, albumin, bilirubin and the vitamin E analogue Trolox. In particular, an about 6 times higher ability, compared to ORAC, to highlight synergistic interactions was observed [26].

Moreover, LOX/RNO assay resulted able to detect an about three-fold higher synergism than ORAC assay among hydrophilic, phenolic and lipophilic antioxidant compounds extracted from durum wheat whole flour, while the TEAC method was ineffective in the same experimental conditions [25].

Performance of LOX/RNO reaction in detecting synergism was also evaluated by measuring interactions within antioxidant compounds in the same type of extract. In particular, comparison was made of inhibition exerted on the reaction rate by phenolic compounds extracted from durum wheat whole flour with that exerted by high-purity ferulic acid preparation. Applying this protocol, LOX/RNO showed an inhibition effectiveness of phenolic mixture about 400-fold higher than that exerted by pure compound, indicating a very strong synergistic action of phenols within the extract, while the same comparison for ORAC and TEAC assays provided an inhibition effectiveness of only about 2.8 and 0.89 [28].

Finally, by using the LOX–FL method, an about eight-fold higher synergism than ORAC assay among human serum and food phenolic antioxidants obtained from the dietary supplement Lisosan G was measured [26].

In conclusion, probably in the light of capability to simultaneously detect several antioxidant functions, LOX-1-based assays show much higher effectiveness than other well-known AC assays in revealing synergism among antioxidant compounds. This has been clearly demonstrated applying different experimental protocols involving mix of different concentrations of antioxidants showing different chemical–physical properties and obtained from very different plant sources. It should be considered that the ability to detect synergism is a very relevant property of an AC assay, since synergistic interactions among food antioxidant compounds are thought to play a critical role in health-promoting effects of some plant foods [63].

In the whole, the main advantages of both LOX-1-based assays can be summarized as follows. Firstly, LOX/RNO and LOX–FL methods employ protocols: (i) technically simple and involving a readily available instrumentation, (ii) able to ensure good reproducibility of results and (iii) easily applicable to either single pure antioxidant compounds or food extracts, both fat-soluble and water-soluble, as well as biological samples (plasma, and serum). Moreover, these assays allow to carry out measurements in experimental conditions resembling those occurring in vivo. In fact, (i) more than one oxidant species having relevant biological significance are involved in LOX-1-based reactions, (ii) the oxidant–antioxidant competition is evaluated under condition of low oxygen concentrations, and (iii) it occurs at the surface of a biological macromolecule (the LOX enzyme) rather than in the bulk phase of the reaction mixture. Moreover, LOX-1-based assays are able to simultaneously evaluate different antioxidant mechanisms, as well as synergistic interactions among antioxidant compounds. In the light of all these properties, LOX-1-based assays may provide a reliable, advanced and comprehensive AC information of natural plant products, potentially more reflecting their in vivo biological effects compared to the most widespread AC assays. Interestingly, the main requirements/criteria proposed by Prior et al. [1] for standardization of AC determination may be considered sufficiently satisfied by both LOX–RNO and LOX–FL methods.

## 3. Evaluation of Human Blood Antioxidant Status after Food Intake

### 3.1. Antioxidant/Oxidant Balance (AOB) Approach and its Application in Short-Term Studies

It should be considered that in vitro AC evaluation may allow for only a merceological characterization of plant food commodities that cannot be related to biological effects of dietary bioactive compounds after food consumption [7,21]. In fact, in vitro AC analysis of food extracts do not provide information about bioavailability of dietary phytochemicals, i.e., the fraction of ingested antioxidant compound reaching the systemic circulation, as well as their in vivo stability, and retention by tissues and in situ reactivity. In the light of these considerations, in 2012, the U.S. Department of Agriculture (USDA) removed its ORAC database for selected foods from its nutrient data laboratory website [22].

In an attempt to obtain a more physiologically relevant information about potential health effects of foods, a new approach has been proposed consisting in associating in vitro AC analysis of foods to ex vivo study of blood antioxidant status after food intake. Assessment of serum/plasma AC after food consumption may take into account bioavailability of dietary phytochemicals, as well as metabolism of food antioxidants, i.e., the possible transformations of phytochemicals by gut microbiota and by intestinal and hepatic metabolism [71]. This may provide an integrated information about the real effect of food antioxidant assumption on blood antioxidant status, which is beyond AC of the ingested food and cannot be deduced by only in vitro AC measurement.

Unfortunately, this approach may also present some weakness, leading to some controversial results. In fact, contrasting results have been obtained from blood AC assessment after consumption of antioxidant-enriched foods, showing only limited AC increase [72,73], as well as no effect [74,75], and in some cases, even an AC decrease [35,75]. It should be also considered that some reports showed no AC change accompanied by a decrease of serum/plasma oxidation level [35,76]. These findings suggest the unsuitability of blood AC measurements alone to derive information on food antioxidant effectiveness and the need to consider also changes of serum oxidative status. In the light of this, aimed at overcoming this weakness, an advancement with respect to measurement of serum antioxidant status was attempted by proposing a novel parameter, named as “Antioxidant/Oxidant Balance” (AOB). It involves evaluation of both antioxidant and oxidant status of blood after food intake and is obtained as a ratio between serum AC and serum oxidant status, evaluated as “Peroxide Level” [AOB = AC/Peroxide Level (PxL)] [34]. It should be outlined that PxL provides an information about serum hydroperoxides resulting highly correlated to that obtained by oxidized low-density lipoproteins assay [34].

Efficacy of the novel AOB approach in highlighting changes in serum antioxidant status after food intake was assessed for the first time by evaluating short-term (up to 4 h) effects of consumption of two antioxidant-enriched pastas, supplemented with bran oleoresin (BO) and bran water (BW) extracts, respectively (see also Table 3), in comparison with a non-supplemented reference (R) pasta [34]. Preliminarily, performance of AOB parameter was verified after intake of two foods, which are expected to induce an opposite effect on serum AC. They included the dietary supplement Lisosan G, able to display high in vitro AC [26,30] (see also Table 3) and glucose, known to induce a pro-oxidant effect [77,78]. In Laus et al. [34], AOB profiles, obtained within 4 h after Lisosan G and glucose assumption by calculating AC_LOX–FL_/PxL, AC_ORAC_/PxL or AC_TEAC_/PxL ratios, are reported in comparison with AC and PxL profiles. While serum AC measurements provided inconsistent results among different assays [79], AOB profiles evaluated both as AC_LOX–FL_/PxL and as AC_ORAC_/PxL or AC_TEAC_/PxL agreed in pointing out a remarkable improvement of serum antioxidant state after Lisosan G intake, as well as a marked decrease due to glucose ingestion [34]. Concerning pastas, all AOB profiles highlighted the expected worsening of serum antioxidant status after R pasta intake similar to glucose; a decreasing trend was also observed after consumption of R pasta together with Lisosan G [34]. Interestingly, BO pasta consumption allowed for a significant improvement of AOB_LOX–FL_, as well as that of AOB_ORAC_ or AOB_TEAC_, similarly to Lisosan G [34]. Contrarily, BW pasta was unable to exert a positive effect on serum, but it even induced an oxidative effect, as did R pasta and glucose.

**Figure 4:**
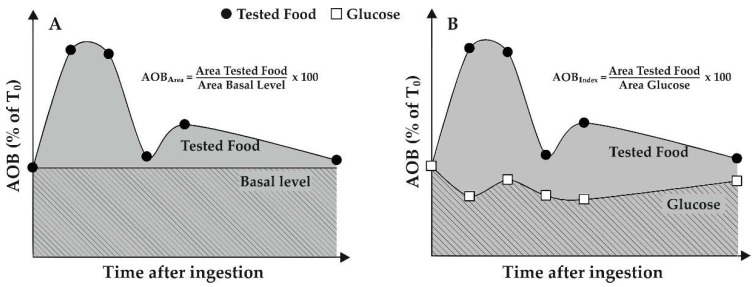
Calculation of AOB-Area and AOB-Index after food consumption. Hypothetical AOB profiles relative to both a generic food and glucose intake are reported. (**A**) AOB-Area is calculated as a ratio (%) between the area under serum AOB (expressed as % of T_0_ value, i.e., value obtained before food intake) profile vs time (grey colored area) and the area below the baseline (crossed area), i.e., area below value at the baseline (T_0_). (**B**) AOB-Index is calculated as a ratio (%) between the area under AOB profile and the area obtained after glucose (50 g) ingestion (crossed area).

In order to facilitate the comparison of short-term effects of food intake on serum AOB, a quantification was proposed involving the evaluation of area under AOB profiles vs time. Two AOB-derived parameters, indicated as AOB-Area and AOB-Index, were proposed [34]. As shown in Figure 4, AOB-Area is obtained as change (%) of area under serum AOB profile vs time of the tested food with respect to the basal area, i.e., area below the value obtained on fasting. AOB-Index is calculated as change (%) of area under the AOB profile with respect to the area obtained after 50 g glucose ingestion, thus taking into account the effect of starch component of pasta (in terms of glucose released from starch digestion).

**Figure 5:**
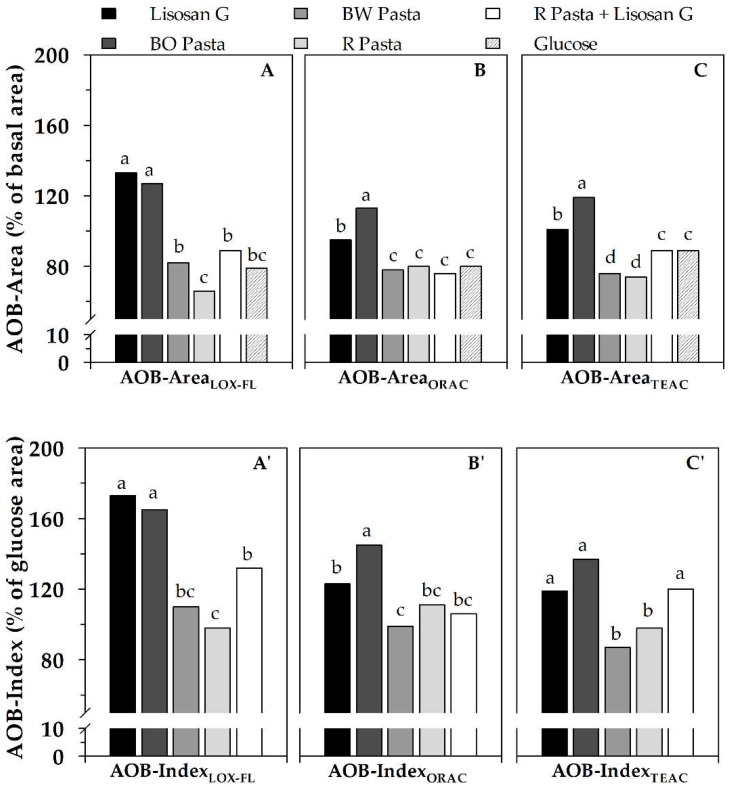
AOB-Area ((**A**), (**B**), (**C**)) and AOB-Index ((**A’**), (**B’**), (**C’**)) of serum, evaluated as AC_LOX–FL_/PxL (AOB_LOX–FL_), AC_ORAC_/PxL (AOB_ORAC_) and AC_TEAC_/PxL (AOB_TEAC_) ratios, after consumption of different foods in seven subjects. Serving sizes were 70 g for pastas, 20 g (fresh weight) for Lisosan G and 50 g for glucose. AOB-Area and AOB-Index values of each tested food represent areas under AOB profile vs time (from 0 to 240 min), expressed as (%) of basal area and (%) of area relative to glucose consumption, respectively (see Figure 4). Data are reported as mean value (*n* = 7 subjects). Within the same graph, different letters indicate significant differences at *p*-value equal to 0.05, according to the Duncan’s test. BO pasta: pasta supplemented with bran oleoresin; R pasta: reference pasta; BW pasta: pasta supplemented with bran water extract. Data are properly re-elaborated from that reported in Laus et al. [34].

In Figure 5, AOB-Area (A–C) and AOB-Index (A’–C’) of serum after consumption of BO, BW and R pastas, in comparison with that relative to intake of Lisosan G, glucose and R pasta added with Lisosan G are reported. The superiority of the antioxidant-rich supplement Lisosan G with respect to the pro-oxidant glucose was clearly highlighted by AOB-Area, regardless of the assay used to measure AC. Moreover, a relevant antioxidant effect on serum, equal to and even higher than that of Lisosan G, was attributed to BO pasta by AOB-Area_LOX–FL_ or AOB-Area_ORAC_ and AOB-Area_TEAC_, respectively. On the contrary, BW pasta exerted a pro-oxidant effect on serum, as indicated by AOB-Area values, obtained as AC_LOX–FL_/PxL and AC_ORAC_/PxL or AC_TEAC_/PxL, equal to and even lower than that of glucose, respectively. A behavior similar to glucose was also observed for R pasta consumed together with Lisosan G. As expected, a worsening equal to and slightly higher than that of glucose was pointed out for the non-supplemented R pasta by AOB-Area determined as AC_LOX–FL_/PxL and AC_ORAC_/PxL or AC_TEAC_/PxL, respectively. The different behavior of BW and BO pastas suggested inefficacy, under the adopted experimental conditions, of hydrophilic/phenolic antioxidants in compensating serum oxidation due to glucose release from starch digestion, as well as the capability of lipophilic compounds of both the counteracting detrimental effect of starch/glucose and improving serum antioxidant status [34].

AOB-Index essentially confirmed AOB-Area data, but it was capable of amplifying the changes (Figure 5A’–C’). In fact, besides confirming the similar behavior of BW and R pastas, AOB-Index_LOX–FL_ highlighted a remarkable beneficial effect on serum antioxidant status of BO pasta and Lisosan G up to about +70% and +65%, respectively, compared to R pasta. Interestingly, a significant improvement of R pasta consumed with Lisosan G compared to R pasta alone was also pointed out by AOB-Index_TEAC_ and AOB-Index_LOX–FL_.

A comparison of AOB responses to these obtained by ex vivo serum AC analysis allows for interesting observations. In fact, as for serum AC measurement, only LOX–FL assay resulted able to discriminate between Lisosan G and glucose, while ORAC and TEAC failed to do this [34,79]. Moreover, only LOX–FL method measured a slightly higher serum AC after BO pasta consumption in comparison to both BW and R pastas, while TEAC and ORAC did not highlight any difference among them [34,79]. If in vitro AC measurements are also considered, LOX–FL, ORAC and TEAC methods agreed only in highlighting a remarkable AC of Lisosan G, but provided contrasting results on cooked pastas [34] (see also Table 3). Overall, these findings indicate suitability and reliability of AOB approach, which is capable of assessing short-term changes of blood antioxidant status after antioxidant-enriched food consumption that cannot be predicted by ex vivo analysis of AC alone, as well as by in vitro measurements of foods. Interestingly, the use of AOB parameter also re-enables ORAC and TEAC assays. In the light of all these observations, AOB parameter appears as a more performing tool than AC assessment for evaluation of effects of food intake on serum antioxidant status.

In conclusion, by applying this innovative approach to short-term evaluation of food consumption, high antioxidant properties of the dietary supplement Lisosan G have been confirmed, as well as the pro-oxidant effect of glucose ingestion. Interestingly, using the same approach, the real health effects of two antioxidant-supplemented pastas have been verified. In particular, AOB has allowed highlighting a strong efficacy in enhancing serum antioxidant status of the pasta supplemented with durum wheat bran oleoresin extract, as well as excluding a possible biological effect of the other putative functional pasta, added with bran phenolic compounds [34].

### 3.2. AOB Approach in Long-Term Studies

The reliability of AOB approach was also assessed in two long-term small pilot studies [35]. In the first study, eight enrolled subjects consumed 250 g/day of cherry puree for three weeks. As for the second study, 11 recruited volunteers participated in “Med-Food Anticancer Program (MFAP)” nutrition education intervention, consisting in a promotion of a balanced Mediterranean diet, involving consumption of several different antioxidant-rich plant foods to obtain an about 50% increased normal daily antioxidant intake [35]. AC measurements were not able to highlight serum AC increase; a 7–9% significant decrease was even measured by LOX–FL and ORAC methods in both studies. On the other hand, a much more evident (about 20%) significant decrease of PxL was found [35]. Interestingly, when analysis moved from AC to AOB approach, both long-term studies were able to highlight very well an improvement of serum antioxidant status (ranging from about +20% to +40%) [35]. This shows reliability of the new approach in highlighting the expected positive changes of serum antioxidant status due to long-term regular dietary intake of antioxidant-rich plant foods. Interestingly, the AOB approach resulted able in revealing health positive effects of whichever kind of dietary antioxidants administered.

In the whole, the AOB approach appears as a more performing tool than both in vitro and ex vivo AC determinations alone. Interestingly, the AOB parameter provides the similar results with whichever assay used to measure AC, although higher performance was obtained by using the novel LOX–FL method, probably due to its capability to provide a more biologically relevant AC information. This good performance of AOB approach depends on the simultaneous evaluation of AC and PxL, which allows accounting for both AC and the AC fraction not directly measureable as consumed to compensate serum oxidation [34,35], and may solve some misleading results obtained by ex vivo serum AC determination alone. Whether a positive effect on blood antioxidant status can be related to a health beneficial effect remains to be established. However, AOB may provide a biologically relevant information, since the maintenance of blood physiological antioxidant status is required to prevent aging-related endothelial dysfunction and consequent cardiovascular disease [80].

It should be also outlined that the AOB approach has been recently applied as a biomarker of blood antioxidant status in ewes fed with *Ascophyllum nodosum* and flaxseed under high ambient temperature [81] and in sheep suffering foot rot and treated with ozone therapy [82], thus demonstrating its high versatility and applicability both in human and animal studies.

## 4. Conclusions

Methodological approaches may have a crucial role in assessment of putative healthful properties of dietary phytochemicals. In fact, the selection of appropriate methodologies is necessary to avoid incorrect and misleading data interpretation about antioxidant properties of foods. With respect to this issue, here, a novel advanced methodological approach has been discussed. It combines: (i) in vitro AC measurements of food extracts by means of innovative assays based on the soybean LOX-1 reactions able to produce several physiological radical species in experimental conditions approaching the cellular ones and to reveal different antioxidant attributes and (ii) ex vivo analysis of blood serum after food consumption, involving both the AC and peroxidation level determination and calculation of AOB parameter as the AC/PxL ratio. In conclusion, this combined in vitro*/*ex vivo study of antioxidant properties of foods using LOX-1-based methodologies appears as a highly performing tool able to provide a more integrated and trustworthy information about potential health value of dietary antioxidants, which is unpredictable by AC analysis alone.

The picture emerging from this advanced approach can be further valorized by evaluation of biological effects of phytochemicals at the cellular/subcellular level.

## Figures and Tables

**Table 1:** List of the widespread assays to evaluate Antioxidant Capacity.

Assay	Main Mechanism	Oxidant	Probe	Detection	Ref.
ORAC	HAT	ROO∙	FL	Fluorescence	[1,7]
DPPH	SET	DPPH∙	DPPH∙	Absorbance	[8,9]
FRAP	SET	Fe^3+^	[Fe(TPTZ)_2_]^2+^	Absorbance	[10]
TEAC	SET	ABTS∙^+^	ABTS^+^	Absorbance	[11,12]
HORAC	HAT	HO∙	FL	Fluorescence	[13]
TRAP	HAT	ROO∙	β-PE	Fluorescence	[14,15]
CUPRAC	SET	Cu^2+^	Neocuproine	Absorbance	[16]
Total Phenolic Assay	SET	FCR	FCR	Absorbance	[17]
Crocin Bleaching	HAT	ROO∙	Crocin	Absorbance	[18]
Chemiluminescence	HAT	H_2_O_2_	Luminol	Fluorescence	[19]

ABTS∙^+^: 2,2′-azino-bis (3-ethylbenzothiazoline-6- sulfonic acid) radical cation; CUPRAC: Cupric Ion Reducing Antioxidant Capacity; DPPH: 2,2-Diphenyl-1-picrylhydrazyl; DPPH∙: DPPH radical; FCR: Folin Ciocalteu Reagent; [Fe(TPTZ)_2_]^2+^: 2,4,6-Tris(2-pyridyl)-*s*-triazine complex; β-PE: β-phycoerythrin; FL: Fluorescein; FRAP: Ferric Ion Reducing Antioxidant Power; HORAC: Hydroxyl (HO) Radicals Averting Capacity; ORAC: Oxygen Radical Absorbance Capacity; ROO∙: peroxylradical generated by 2,2′-Azobis(2-methylpropionamidine) dihydrochloride; TEAC: Trolox Equivalent Antioxidant Capacity; TRAP: Total Radical-trapping Antioxidant Parameter.

**Table 2:** Effects of different antioxidant compounds on LOX/RNO and LOX–FL reactions.

Compound	K_i_^a^ or IC_50_^b^	Antioxidant/Trolox ^c^
**LOX/RNO Reaction**		
Trolox	7.0 ± 1.1 mM ^a,d^	1.00
Resveratrol	1.7 ± 0.2 mM ^a,d^	4.57 ± 0.33 ^e^
Ferulic acid	10.7 ± 2.1 mM ^a,d^	0.99 ± 0.11^e^
Gallic acid	6.7 ± 1.3 mM ^a,d^	0.61 ± 0.05 ^e^
Apigenin	3.4 ± 0.5 mM ^a,d^	1.49 ± 0.09 ^e^
Catechin	13.6 ± 1.8 mM ^a,d^	0.42 ± 0.03 ^e^
l-Ascorbic acid	14.0 ± 1.9 mM ^a,d^	0.83 ± 0.09 ^e^
Glutathione	19.7 ± 3.2 mM ^b,d^	n.d.
α-tocopherol	1.1 ± 0.1 mM ^a,d^	3.43 ± 0.25 ^e^
β-carotene	7.8 ± 0.9 μM ^b,d^	n.d.
**LOX–FL Reaction**		
Trolox	5 ± 0.6 μM ^a^; 26 ± 2 μM ^b,d^	1.00
Albumin	25 ± 2 μM ^b,d^	1.04 ± 0.08 ^e,f^
Bilirubin	7.6 ± 0.6 μM ^b,d^	3.43 ± 0.28 ^e,f^
l-Ascorbic acid	1360 ± 10 μM ^b,d^	0.02 ± 0.002 ^e,f^
Uric acid	40 ± 2 μM ^b,d^	0.66 ± 0.04 ^e,f^

^a^ Ki values were obtained by measuring reaction rates at different RNO or FL and antioxidant concentrations; ^b^ IC_50_ values represent the antioxidant concentration able to make half the rates of LOX/RNO (or LOX-mediated oxodiene generation in the case of glutathione) or LOX–FL reactions; ^c^ ratio between the gradient of the plot reporting the decrease of the rate (%) of LOX/RNO or LOX–FL reaction as a function of the antioxidant concentration and the gradient of the same plot relative to Trolox; ^d^ mean value ± standard deviation (*n* = 4); ^e^ mean value ± standard error (*n* = 4); ^f^ unpublished data; n.d. = not determined. Adapted from Pastore et al. [25] and Soccio et al. [26].

**Table 3:** Antioxidant Capacities of different plant samples as evaluated by LOX/RNO and/or LOX–FL methods in comparison with TEAC and ORAC assays.

Plant Matrices and Derived Products	Extract	AC (µmol Trolox eq./g Dry Weight)			Ref.
		LOX/RNO and/or LOX–FL ^a^	ORAC	TEAC	
Whole grains of durum wheat (*Triticum durum* Desf., cv. Simeto)	H	102 ± 4.8 ^b^		3.00 ± 0.05 ^b^	[28]
	L	46.3 ± 1.1 ^b^		0.34 ± 0.01 ^b^	
	FSP	41.1 ± 9.7 ^b^			
	IBP	1137 ± 65 ^b^		6.23 ± 0.12 ^b^	
Whole grains of durum wheat (*Triticum durum* Desf., cv. Ofanto)	H	116 ± 9 ^b^	19.3 ± 2.1	2.98 ± 0.09 ^b^	[25,28]
	L	57.3 ± 1.1 ^b^	2.26 ± 0.17	0.35 ± 0.01 ^b^	
	FSP	133 ± 44 ^b^			
	IBP	1336 ± 44 ^b^	14.7 ± 2.0	6.00 ± 0.20 ^b^	
Whole grains of durum wheat (*Triticum durum* Desf., cv. Adamello)	H	106 ± 8	17.3 ± 1.5	5.13 ± 0.40	[29]
	L	73.3 ± 11.9	1.46 ± 0.06	0.24 ± 0.04	
	FSP	47.2 ± 0.8	3.42 ± 0.16	0.89 ± 0.02	
	IBP	800 ± 12	13.5 ± 1.5	6.67 ± 0.14	
Whole grains of bread wheat (*Triticum aestivum* L., cv. Bolero)	H	128 ± 10	32.5 ± 3.5	5.34 ± 0.10	[57,58]
	L	146 ± 14	15.5 ± 0.9	0.19 ± 0.02	
	FSP	54.0 ± 2.0	2.51 ± 0.11	0.97 ± 0.03	
	IBP	256 ± 8	8.28 ± 1.07	3.92 ± 0.21	
Whole grains of naked einkorn (*Triticum monococcum* L. ssp. *sinskajae*)	H	115 ± 12	4.45 ± 0.16	5.61 ± 0.09	[57,58]
	L	210 ± 8	2.83 ± 0.06	0.58 ± 0.02	
	FSP	74.2 ± 2.3	2.42 ± 0.12	0.73 ± 0.03	
	IBP	1081 ± 22	1.93 ± 0.07	0.63 ± 0.01	
Whole grains of hulled einkorn (*Triticum monococcum* L. ssp. *monococcum*)	H	72.3 ± 3.8	3.59 ± 0.02	5.76 ± 0.30	[57,58]
	L	148 ± 11	2.95 ± 0.25	0.40 ± 0.05	
	FSP	263 ± 6	1.70 ± 0.01	0.51 ± 0.01	
	IBP	2008 ± 38	12.0 ± 1.0	4.95 ± 0.17	
Whole grains of emmer (*Triticum dicoccum* Schübler, cv. Molise Colli)	H	360 ± 65	54.6 ± 13.0	5.84 ± 0.12	[29]
	L	141 ± 11	0.88 ± 0.03	0.20 ± 0.01	
	FSP	14.2 ± 0.7	4.25 ± 0.41	0.75 ± 0.04	
	IBP	1239 ± 21	26.0 ± 0.4	1.61 ± 0.09	
Whole grains of spelt (*Triticum spelta* L., cv. Altgold Rotkorn)	H	256 ± 11	22.5 ± 1.7	5.23 ± 0.15	[57,58]
	L	104 ± 12	2.27 ± 0.02	0.80 ± 0.02	
	FSP	186 ± 4	2.51 ± 0.07	0.66 ± 0.02	
	IBP	1764 ± 25	5.84 ± 0.25	1.50 ± 0.05	
Whole grains of finger millet (*Eleusine coracana* L. Gaertn.)	H	565 ± 5		25 ± 0.1	[59,60,61]
Whole grains of teff (*Eragrostis tef* (Zucc.) Trotter)	H	256 ± 8		2 ± 0.1	[59,60,61]
Whole grains of buckwheat (*Fagopyrum esculentum* Moench)	H	82 ± 9.8		16 ± 1.2	[60,61]
Whole grains of amaranth (*Amaranthus* spp.)	H	64 ± 1		3 ± 0.1	[59,60,61]
Saponin-free grains of quinoa (*Chenopodium quinoa* Willd., cv. Real)	H	138 ± 11	37 ± 1	12.8 ± 0.5	[29]
	L	130 ± 6	0.38 ± 0.03	0.33 ± 0.03	
	FSP	81 ± 4	5.75 ± 0.23	1.67 ± 0.06	
	IBP	428 ± 4	4.89 ± 0.15	3.72 ± 0.17	
Durum wheat bran	BW	749 ± 50 (12.5 ± 0.7 ^a^) ^f^	48 ± 4 ^f^	18.9 ± 1.3 ^f^	
Lisosan G (nutritional supplement)	H	1576 ± 427 (81.7 ± 4.7 ^a^)	123 ± 6	48 ± 3	[26,30]
	L	258 ± 2 (0.56 ± 0.06 ^a^)	1.3 ± 0.02	3.7 ± 0.78	
	FSP	83 ± 2 (4.3 ± 0.4 ^a^)	25.6 ± 0.7	7.1 ± 1.2	
	IBP	1294 ± 24 (7.1 ± 0.1 ^a^)	56.6 ± 4.8	29.5 ± 2.1	
Apple (*Malus domestica* Borkh.) fiber	H	333 ± 23		29 ± 2	[60,61]
Coffee silverskin	H	1773 ± 108		27 ± 0.3	[60,61]
Durum wheat semolina pasta	H	2.55 ± 0.09 ^a^	5.15 ± 0.55	2.29 ± 0.20	[34]
	L	0.41 ± 0.04 ^a^	1.11 ± 0.11	0.14 ± 0.02	
	FSP	0.34 ± 0.02 ^a^	1.46 ± 0.16	0.20 ± 0.03	
Durum wheat semolina pasta supplemented with durum wheat bran oleoresin extract	H	2.93 ± 0.11 ^a^	6.03 ± 0.49	2.13 ± 0.1	[34]
	L	1.58 ± 0.1 ^a^	0.86 ± 0.11	0.19 ± 0.003	
	FSP	0.29 ± 0.01 ^a^	1.69 ± 0.19	0.19 ± 0.01	
Durum wheat semolina pasta supplemented with durum wheat bran water extract	H	2.39 ± 0.1 ^a^	4.56 ± 0.25	2.34 ± 0.12	[34]
	L	0.35 ± 0.02 ^a^	1.13 ± 0.08	0.15 ± 0.01	
	FSP	0.92 ± 0.03 ^a^	1.50 ± 0.03	0.29 ± 0.01	
Food-grade resveratrol (98%) from Japanese knotweed (*Polygonum cuspidatum* Siebold et Zucc.) root	-	28.2 ± 0.6 ^c^		6.44 ± 0.71 ^c^	[31]
Food-grade quercetin (98%) from Japanese pagoda tree *(Sophora japonica* L.) flower buds	-	12.7 ± 0.5 ^c^		5.87 ± 0.50 ^c^	[31]
Food-grade catechins (50%) from green tea (*Camellia sinensis* (L.) Kuntze) leaf	-	29.8 ± 0.7 ^c^		3.15 ± 0.45 ^c^	[31]
Food-grade lycopene (15%) from tomato (*Solanum lycopersicum* L.) fruit	-	215 ± 1.5 ^c^		2.45 ± 0.30 ^c^	[31]
OLIPLUS^®^, olive (*Olea europaea* L.) extract containing polyphenols (45%)	-	24.0 ± 1.0 ^c^		1.69 ± 0.30 ^c^	[31]
Extra virgin olive oil (*Olea europaea* L., cv. Cima di Mola)	-	4030 ± 400 ^d^	17.3 ± 1.8 ^d^	4.4 ± 0.2 ^d^	u.d.
Extra virgin olive oil (*Olea europaea* L., cv. Coratina)	-	2800 ± 160 ^d^	26.0 ± 2.7 ^d^	3.9 ± 0.3 ^d^	u.d.
Extra virgin olive oil (*Olea europaea* L., cv. Peranzana)	-	2770 ± 10 ^d^	14.2 ± 0.1 ^d^	2.01 ± 0.07 ^d^	u.d.
Red wine (*Vitis vinifera* L., cv. Negramaro)	-	8.6 ± 0.2 ^a,d^	59.8 ± 5.8 ^d^	24.4 ± 1.3 ^d^	u.d.
Red wine (*Vitis vinifera* L., cv. Nero di Troia)	-	10.7 ± 0.3 ^a,d^	47.1 ± 2.8 ^d^	36.6 ± 0.9 ^d^	u.d.
Red wine (*Vitis vinifera* L., cv. Primitivo)	-	8.0 ± 0.3 ^a,d^	47.6 ± 6.0 ^d^	32.6 ± 1.3 ^d^	u.d.
Puree from cherry (*Prunus avium* L., cv. Ferrovia) fruit	FSP	5.1 ± 0.2 ^a,e^	19.1 ± 0.1 ^e^	6.5 ± 0.1 ^e^	[35]
Peach (*Prunus persica* L., cv. Redhaven) fruit	C	155 ± 10 ^e^	0.33 ± 0.04 ^e^	0.082 ± 0.002 ^e^	[32]
Peach (*Prunus persica* L., cv. Armking) fruit	C	125 ± 10 ^e^	0.44 ± 0.06 ^e^	0.068 ± 0.001 ^e^	[32]
Peach (*Prunus persica* L., cv. Silverking) fruit	C	9.6 ± 3.5 ^e^	0.21 ± 0.04 ^e^	0.025 ± 0.001 ^e^	[32]
Peach (*Prunus persica* L., cv. Caldesi 2000) fruit	C	10.5 ± 1.2 ^e^	0.11 ± 0.01 ^e^	0.018 ± 0.0001 ^e^	[32]
Peach (*Prunus persica* L., cv. IFF331) fruit	C	9.8 ± 1.5 ^e^	0.13 ± 0.01 ^e^	0.020 ± 0.0001 ^e^	[32]
Tomato (*Solanum lycopersicum* L.) fruit	H	3.79 ± 0.84 ^a^	64.4 ± 10.7	38.3 ± 4.0	u.d.
	L	11.25 ± 5.24 ^a^	41.6 ± 15.4	3.88 ± 0.87	
Juice of pomegranate (*Punica granatum* L.) fruit	-	36.2 ± 5.4 ^a,d^	14.6 ± 3.1 ^d^	45.8 ± 1.5 ^d^	u.d.

H: hydrophilic extract; L: lipophilic extract; FSP: free-soluble phenolic extract; IBP: insoluble-bound phenolic extract; C: carotenoid-enriched extract; u.d.: unpublished data. Olive oil, red wine and pomegranate juice samples were analyzed without extraction. ^a^ evaluated by the LOX–FL method; ^b^ mean value (± SE) of four whole grain samples obtained from plants subjected to different growing conditions (for details, see Laus et al. [28]); ^c^ μmol Trolox eq./mg fresh weight; ^d^ μmol Trolox eq./mL; ^e^ μmol Trolox eq./g fresh weight.

**Table 4:** Synergism among antioxidant compounds evaluated by LOX/RNO and LOX–FL methods in comparison with ORAC and TEAC assays.

	LOX/RNO ^a^ or LOX–FL ^b^	ORAC	TEAC	Ref.
	Among Different Pure Compounds (% Change) ^c^			
Mix of the food-grade extracts resveratrol, quercetin, OLIPLUS^®^, catechin and lycopene	+570 ***^,a^	-	+30 **	[31]
Mix of ascorbic acid, Trolox, bilirubin, uric acid and albumin	+74 ***^,b^	+12 ^n.s.^	-	[26]
	**Among different extracts (% change) ^c^**			
Mix of hydrophilic, lipophilic and insoluble-bound phenolic extracts from durum wheat whole flour	+108 **^,a^	+39 *	−32 *	[25]
	**Among phenols in the same extract (time fold change) ^d^**			
Insoluble-bound phenols from durum wheat whole flour	410 ^a^	2.8	0.89	[28]
	**Among human serum and free-soluble phenols (% change) ^c^**			
Mix of human blood serum and free-soluble phenolic extract from Lisosan G	+124 ***^,b^	+16 *	-	[26]

^a^ evaluated by the LOX/RNO method; ^b^ evaluated by the LOX–FL method; ^c^ change (%) of AC of the mix with respect to the sum of AC of each individual compounds (or extracts); ^d^ time fold change of IC_50_ values of pure ferulic acid with respect to that of phenolic extract. *, *p* ≤ 0.05; **, *p* ≤ 0.01; ***, *p* ≤ 0.001; n.s. = not significant, according to the Student’s *t*-test.
